# Post-Transcriptional Regulation of Immune Responses and Inflammatory Diseases by RNA-Binding ZFP36 Family Proteins

**DOI:** 10.3389/fimmu.2021.711633

**Published:** 2021-07-01

**Authors:** Sohei Makita, Hiroaki Takatori, Hiroshi Nakajima

**Affiliations:** ^1^ Department of Allergy and Clinical Immunology, Graduate School of Medicine, Chiba University, Chiba, Japan; ^2^ Department of Rheumatology, Hamamatsu Medical Center, Hamamatsu, Japan

**Keywords:** tristetraprolin, zinc finger protein 36, zinc finger protein 36-like 1, zinc finger protein 36-like 2, RNA-binding protein, untranslated region, AU-rich element

## Abstract

Post-transcriptional regulation is involved in the regulation of many inflammatory genes. Zinc finger protein 36 (ZFP36) family proteins are RNA-binding proteins involved in messenger RNA (mRNA) metabolism pathways. The ZFP36 family is composed of ZFP36 (also known as tristetraprolin, TTP), ZFP36L1, ZFP36L2, and ZFP36L3 (only in rodents). The ZFP36 family proteins contain two tandemly repeated CCCH-type zinc-finger motifs, bind to adenine uridine-rich elements in the 3’-untranslated regions (3’ UTR) of specific mRNA, and lead to target mRNA decay. Although the ZFP36 family members are structurally similar, they are known to play distinct functions and regulate different target mRNAs, probably due to their cell-type-specific expression patterns. For instance, ZFP36 has been well-known to function as an anti-inflammatory modulator in murine models of systemic inflammatory diseases by down-regulating the production of various pro-inflammatory cytokines, including TNF-α. Meanwhile, ZFP36L1 is required for the maintenance of the marginal-zone B cell compartment. Recently, we found that ZFP36L2 reduces the expression of *Ikzf2* (encoding HELIOS) and suppresses regulatory T cell function. This review summarizes the current understanding of the post-transcriptional regulation of immunological responses and inflammatory diseases by RNA-binding ZFP36 family proteins.

## Introduction

For many years, the importance of post-transcriptional regulation of mRNAs has not been fully recognized in the immune system. However, with the advance in functional analyses of RNA-binding proteins (RBPs), the importance of post-transcriptional regulation in immune system regulation has come to the fore. RBPs are critical effectors of gene expression of many genes and form regulatory networks to maintain cell homeostasis. RBPs recognize target RNA with the RNA-recognition domain (1). RBPs also have binding domains with other proteins, and these interactions enable them to fulfill their regulatory functions (2).

Recent analyses have shown that RBPs are remarkably involved in regulating various cell type-specific functions (3). Among RBPs, ZFP36 family proteins including ZFP36, known as tristetraprolin **(**TTP), are characterized by the presence of one or more CCCH-type zinc finger domain(s) that contain three cysteines (C) and one histidine (H) residues. ZFP36 family proteins bind to adenylate-uridylate-rich elements (AREs) in the 3’-untranslated region (3’ UTR) of a target mRNA, leading to the decay of the mRNA (4). Although the ZFP36 family members are structurally similar, they play different roles and regulate different target mRNAs, probably due to their cell type-specific expression patterns (5). For instance, ZFP36 plays a significant role in regulating immune responses and inflammatory diseases by inhibiting the production of various inflammatory cytokines such as TNF-α in macrophages (6).

ZFP36L1 is known to be required for the maintenance of the marginal zone B cell compartment by limiting the expression of the transcription factors such as Kruppel-like factor 2 (KLF2) and interferon regulatory factor 8 (IRF8) (7). We have recently reported that ZFP36L2 down-regulates the expression of *Ikzf2* (encoding HELIOS) and suppresses the function of induced regulatory T cells (iTregs) (8). In this review, we discuss our current understanding of post-transcriptional regulation in immune responses by RNA-binding ZFP36 family proteins. We also discuss the control of those protein expressions as potential therapeutic strategies for human inflammatory diseases.

## RNA-Binding Proteins Are Involved in Post-Transcriptional Regulation

RBPs recognize cis-elements or specific structures in the 5’ UTR, 3’ UTR, or intron of mRNA at each step of RNA metabolism (9). Adenylate-uridylate-rich elements (AU-rich elements; AREs) characterized by AUUUA nucleotide repeats are present in the 3’ UTRs of many cytokines, chemokines, and proto-oncogenes (3), and ARE-binding RBPs, including ZFP36, human antigen R (HuR)/ELAVL1, AU-rich RNA binding factor 1 (AUF1), T-cell interleukin-1 (TIA-1)/TIA-associated protein (TIAR), and KH-type splicing regulatory protein (KSRP), regulate the degradation and translation of target mRNA (3). In contrast, several other RBPs such as Roquin, regulatory RNase (Regnase), and AT-rich interactive domain-containing protein 5a (Arid5a) recognize the stem-loop structure of the 3’ UTR (3). Thus, RBPs can interact with specific RNA sequences and structures and interact with them to regulate target mRNAs positively or negatively (3).

## ZFP36 Family Members Are Critical for Post-Transcriptional Regulation

ZFP36 family is composed of three proteins (ZFP36 (TTP), ZFP36L1, and ZFP36L2) in humans and most other mammals, while the fourth subtype, ZFP36L3, is expressed in the yolk sac and placenta of rodents (10). The ZFP36 family members are known to have three essential domains: An N-terminal nuclear export sequence (NES), a central tandem Cys-Cys-Cys-His (CCCH) zinc finger domain, and a C-terminal CNOT1 binding domain (9). Although ZFP36 family members are structurally similar to each other, each ZFP36 family member is thought to have different functions, as it has been shown in both immune and non-immune cells that each ZFP36 family member is expressed in different cell-type and is differently controlled upon stimulation (8, 11).

**Figure 1:**
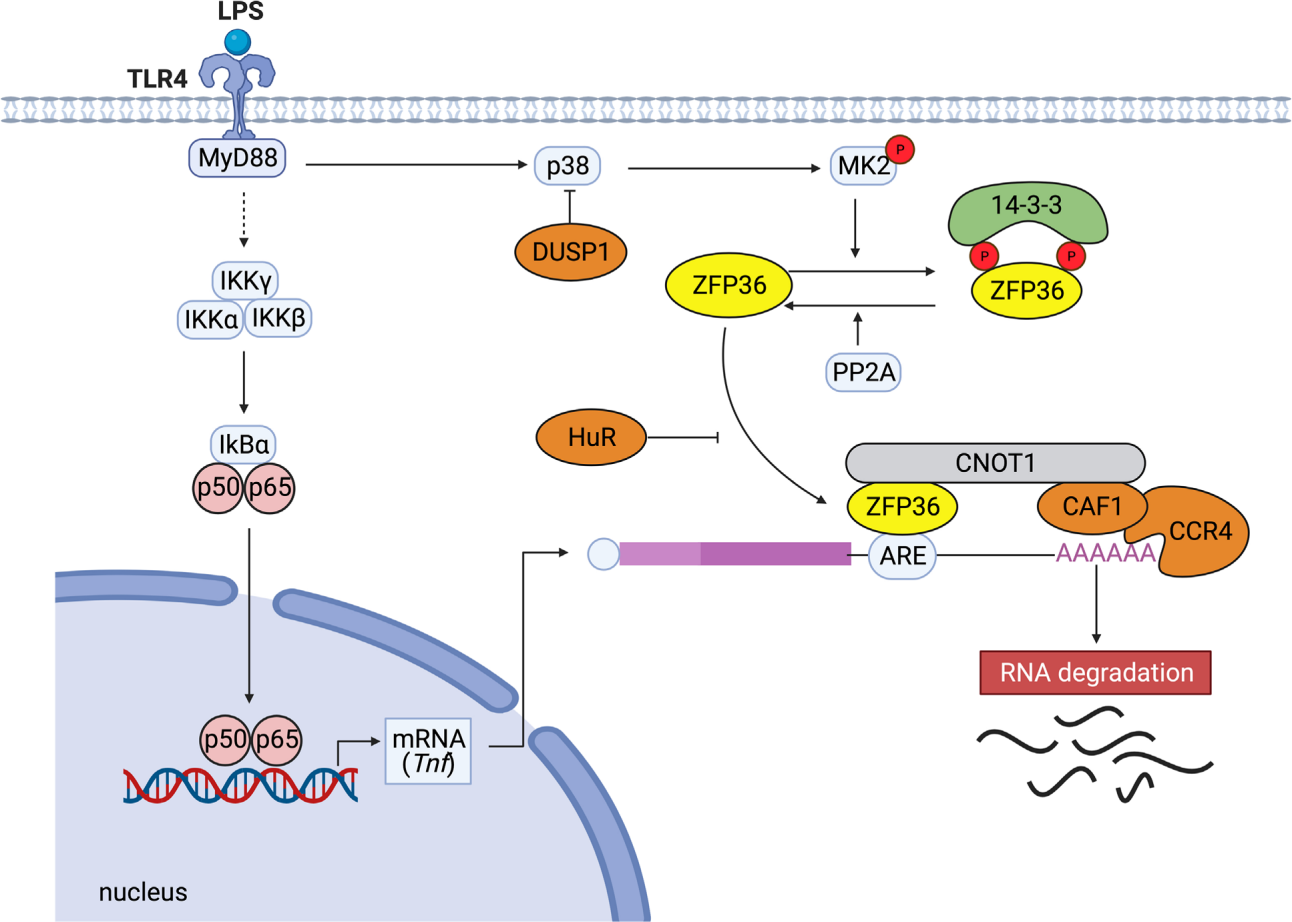
Typical post-transcriptional regulation by ZFP36. When LPS activates TLR4, the downstream NF-κB kinase (IKK) complexes (IKKγ, IKKα, IKKβ) are activated. Subsequently, IκBα is phosphorylated and degraded. The released NF-κB migrates to the nucleus and activates the expression of genes such as TNF. ZFP36 binds to the ARE in the 3’ UTR of *Tnf* mRNA and promotes the decay of the target mRNA by recruiting the CCR4-CAF1-CNOT1 complex. ZFP36 binds directly to the central domain of CNOT1, the core subunit of the CCR4-NOT complex. Conversely, the binding of stabilizing proteins such as human antigen R (HuR) that compete with destabilizing factors inhibits ARE-mediated RNA degradation. p38 MAPK activates MK2, which phosphorylates two serine residues of ZFP36 (S60 and S186 in humans, S52 and S178 in mice). Dual-specificity phosphatase DUSP1 dephosphorylates p38 MAPK. Phosphorylation of ZFP36 promotes its binding to 14-3-3 proteins, resulting in stabilization of target mRNAs. Serine/threonine PP2A dephosphorylates ZFP36 and releases 14-3-3 proteins from ZFP36.

Among ZFP36 family members, the molecular mechanisms of the post-transcriptional regulation are most intensively investigated for ZFP36 (9). The C-terminal motif of ZFP36 binds directly to the central domain of CNOT1, which is the core subunit of the CCR4-NOT complex, and the ZFP36-CCR4-NOT complex plays a crucial role in ZFP36-mediated deadenylation of target mRNAs (12). The deadenylation is thought to be important for rapid mRNA degradation and to be induced in small nests of the cytoplasm (called processing bodies) containing many enzymes (13). Under stress conditions, ZFP36-bound mRNAs are recruited to stress granules, and the translation repressor, TIA-1, prevents translation in stress granules (14). In addition, ZFP36 has been shown to facilitate the degradation of selected mRNAs by transporting them from stress granules to processing bodies (15, 16). Taken together, although the precise mechanism of mRNA turnover by ZFP36 is still unclear, various factors such as the CCR4-NOT complex seem to be essential for the regulation of ZFP36-mediated decay of mRNAs (**Figure 1**).

## Regulation of the Expression and Function of ZFP36

With respect to the molecular mechanisms to regulate ZFP36 expression, it has been shown that ZFP36 autoregulates its expression *via* interaction with AREs in 3’ UTR of its mRNA (17). Experimental deletion of ARE from *Zfp36* mRNA has been shown to free ZFP36 from autoinhibition or repression by other ARE-binding proteins and increase the abundance of ZFP36 (18). Regarding the second mechanism to regulate ZFP36 function, phosphorylation is reported to be involved in the stabilization and inactivation of ZFP36. ZFP36 is phosphorylated by multiple kinases such as ERK, p38 MAPK, JNK, and AKT (19). MAPK-activated protein kinase 2 (MK2) is activated by p38 MAPK and phosphorylates ZFP36 at two serine residues (S60 and S186 in humans, and S52 and S178 in mice) (20, 21). Phosphorylated ZFP36 is more stable than unphosphorylated ZFP36, and the phosphorylated ZFP36 accumulates until p38 MAPK activity is reduced (22, 23). Moreover, the phosphorylation of ZFP36 promotes its binding to 14-3-3 proteins, and the resultant ZFP36-14-3-3 complex does not recruit the CNOT deadenylase complex (21, 22). Therefore, phosphorylated ZFP36 seems to lose its ability to degrade mRNA.

Dual-specificity phosphatase 1 (DUSP1) is known to dephosphorylate and inactivate MAPK superfamily members such as JNKs, p38a, and p38b MAPKs, and then DUSP1 and ZFP36 cooperate to regulate inflammation (23). The loss of DUSP1 leads to ZFP36 phosphorylation and accumulation of inactive ZFP36, and the production of TNF-α and IL-10 is enhanced in Dusp1-deficient bone marrow-derived macrophages (23). Moreover, protein phosphatase 2A (PP2A) activation has been shown to induce dephosphorylation and activation of ZFP36 (21).

In terms of the other mechanisms preventing ZFP36 function, HuR competes with ZFP36 for the AREs in the 3’ UTR of *Il6* mRNA and stabilizes it (24). In addition, ZFP36 is polyubiquitinated by TNF receptor-associated factor 2 (TRAF2), and the polyubiquitination appears to be specifically necessary for its function for JNK activation (25). These studies suggest that multiple mechanisms in immune responses regulate the expression and function of ZFP36, and various kinases affect the activation and stability of ZFP36 in response to different environmental cues (**Figure 1**).

## ZFP36 Controls Various Immune Responses

It is well known that mRNAs encoding cytokines such as TNF-α have short half-lives and decay *via* AREs (26). ZFP36 down-regulates TNF-α production by directly binding to the ARE in the 3’ UTR of *Tnf* mRNA and promoting *Tnf* mRNA decay by recruiting the CCR4-NOT deadenylase complex (6). Meanwhile, ZFP36 expression is induced by TNF-α-mediated signaling. Thus, ZFP36 acts as one component of a negative feedback loop that regulates TNF-α production by destabilizing *Tnf* mRNA (27). In accordance with this finding, ZFP36-deficient mice develop a complex syndrome of inflammatory arthritis, dermatitis, cachexia, autoimmunity, and bone marrow hyperplasia, which resemble the phenotypes due to excessive TNF-α production *in vivo* just like the phenomena observed in TNF-transgenic mice (28, 29).

Not only TNF-α but also IL-6 is well-known to be a multifunctional pro-inflammatory cytokine that plays a critical role in various diseases, and its expression is tightly regulated at both the transcriptional and post-transcriptional levels (4). There are five AREs in the 3’ UTR of murine *Il6* mRNA, and ZFP36 is shown to bind to ARE2, ARE3, and ARE4 in the 3’ UTR region to promote *Il6* mRNA degradation (30).

Surprisingly, the mRNA of IL-10, which is one of the representative anti-inflammatory cytokines, was also identified as a target of ZFP36. Consistent with studies regarding TNF-α and IL-6, *Il10* mRNA degradation was induced by the binding of ZFP36 to ARE in its 3’ UTR (31). Furthermore, IL-10 induces the ZFP36 expression in macrophages by activating STAT3 (32). Thus, IL-10-mediated ZFP36 induction seems a part of the negative feedback loop to regulate IL-10 production to terminate anti-inflammatory signals. Interestingly, Schaljo et al. have reported that IL-10 reduces TNF-α expression in LPS-activated bone marrow-derived murine macrophages in part through the induction of ZFP36 (33). Together, it is suggested that ZFP36-mediated post-transcriptional regulatory mechanisms control both the initiation and resolution of inflammatory responses in multiple mechanisms.

With respect to the roles of ZFP36 in T cell-mediated immune responses, Moore et al. have recently shown that using a lymphocytic choriomeningitis virus (LCMV) infection model, virus-specific expansion and recession of T cells is accelerated, and LCMV clearance is enhanced by the absence of ZFP36 (34), suggesting that ZFP36 restrains T cells and slows down the immune responses.

Taken together, ZFP36 regulates immune responses in various immune cells through many mechanisms.

## The Roles of ZFP36L1 and ZFP36L2 in Immune Responses

Similar to ZFP36, ZFP36L1 interacts with AREs in the 3’ UTR of mRNAs to attenuate the expression of the corresponding genes (35). Regarding the role of ZFP36L1 in post-transcriptional regulation, it has recently been demonstrated that ZFP36L1 expressed in B cells has an essential function in maintaining a population of marginal zone B cells by limiting the expression of KLF2 and IRF8 (7). Although the precise roles of ZFP36L1 in germinal center responses and immune memory remain unclear, it has been reported that ZFP36L1 expressed in B cells promotes the migration of antibody-secreting cells from secondary lymphoid organs to survival niches in the bone marrow by restricting the expression of G protein-coupled receptor kinase 2 (GRK2) and integrin chains α4 and β1, facilitating the long-term establishment of antibody-secreting cells (36).

In developing B cells, because the expression of recombination activating gene 2 (RAG2) protein is restricted to the G0-G1 phase of the cell cycle (37–39), quiescence is essential for promoting variable-diversity-joining (VDJ) recombination. Recently, Galloway et al. have shown that in developing B cells, both ZFP36L1 and ZFP36L2 are important for maintaining quiescence before expressing pre-B cell receptor (pre-BCR) and for the re-establishment of quiescence after expansion by the pre-BCR (40). Importantly, double-deficiency of ZFP36L1 and ZFP36L2 in T-cell lineage in mice causes the arrest of thymopoiesis at the double-negative stage and develops T cell acute lymphoblastic leukemia (T-ALL) due to aberrant activation of Notch signaling (41). In contrast, the single deletion of ZFP36L1 or ZFP36L2 in T-cell lineage does not result in T-ALL (41). These findings suggest that ZFP36L1 and ZFP36L2 play both redundant and non-redundant roles in lymphocyte differentiation.

**Figure 2:**
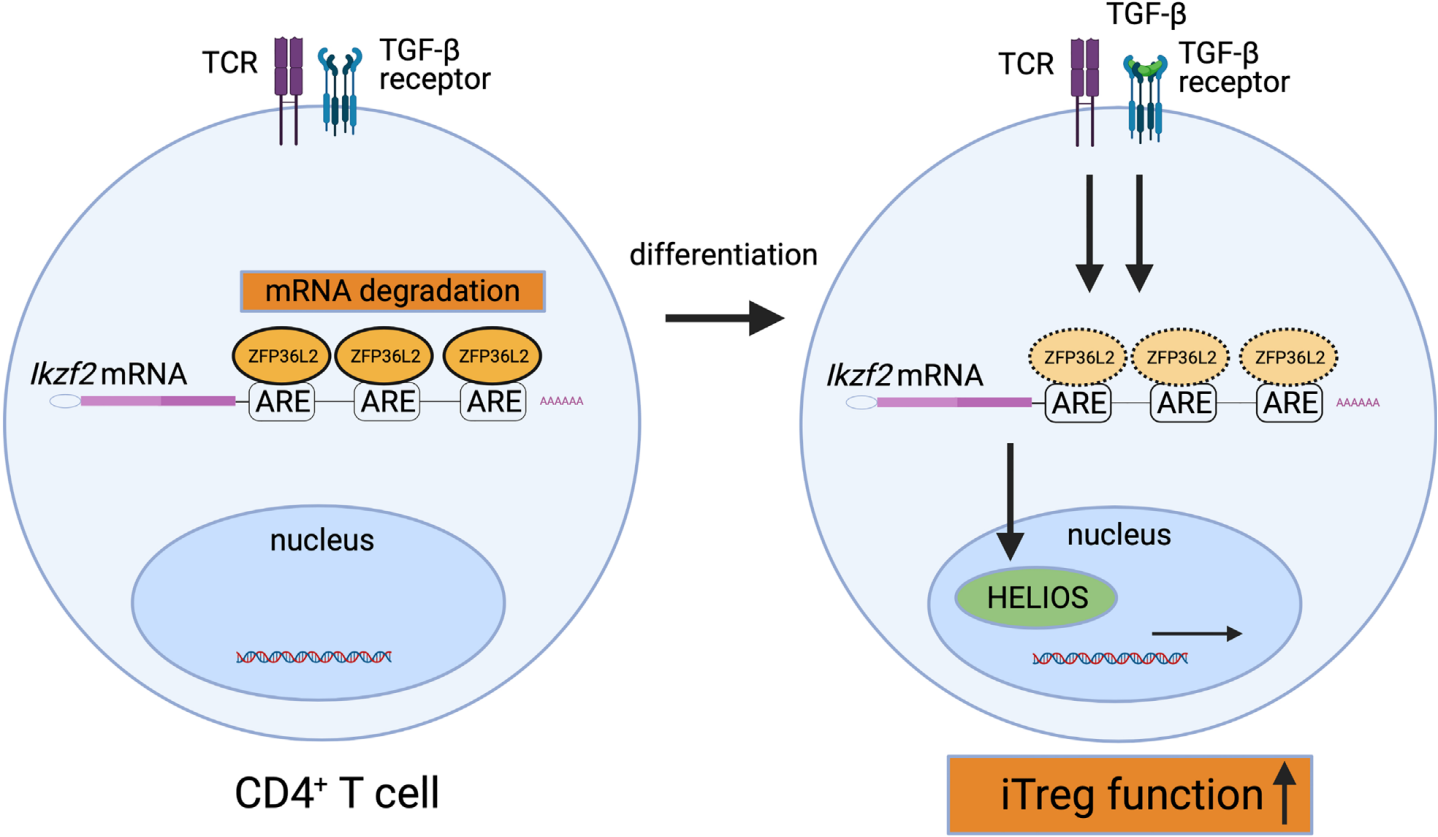
Suppression of HELIOS expression by ZFP36L2. ZFP36L2 is highly expressed in naïve CD4^+^ T cells and can degrade *Ikzf2* mRNAs by binding ARE in the 3’ UTR. Upon TCR stimulation, ZFP36L2 expression is rapidly reduced. Consequently, the transcribed *Ikzf2* mRNA is stabilized, HELIOS is firmly produced, CD4^+^ T cells differentiate and mature into iTregs possessing sufficient suppressive capacity.

How ZFP36L2 alters the function of T cells is not fully understood yet. We have recently shown that ZFP36L2 is highly expressed in naive CD4^+^ T cells, and ZFP36L2 expression in CD4^+^ T cells is rapidly reduced by the stimulation *via* the T cell receptor (8). In addition, we found that ZFP36L2 expression levels in iTregs are significantly lower than those in naive CD4^+^ T cells (8). Moreover, we found that ZFP36L2 directly binds to AREs in 3’ UTR of *Ikzf2* mRNA, resulting in its degradation of *Ikzf2* mRNA and down-regulation of iTreg function (**Figure 2**) (8). These results indicate that ZFP36L2 also promotes post-transcriptional regulation of immune responses and regulates immune cell function.

## Clinical Implication of ZFP36 Family Proteins in Human Inflammatory Diseases

Genome-wide association studies (GWAS) have highlighted the association of *ZFP36 *family members with pathogenic mechanisms in various autoimmune diseases. Twenty-eight single-nucleotide polymorphisms (SNPs) in the *ZFP36* gene were found in patients with autoimmune disorders such as rheumatoid arthritis (RA), psoriasis, multiple sclerosis (MS), and juvenile idiopathic arthritis (JIA) (42).

Interestingly, one SNP called ZFP36*8 variant has been shown to be significantly associated with RA in African Americans (42). Suzuki et al. have reported that compared with AA/AG genotypes, GG genotype in *ZFP36* promoter region SNP, in which promoter activity is lower than that with AA/AG genotypes, is associated with age at onset, duration, disease progression, and infliximab usage in Japanese RA patients (43).

It is not yet clear how ZFP36 is involved in the pathogenesis of human diseases.　It has been reported that ZFP36 is highly expressed in synovial tissues of RA patients and inflamed mucosal tissues of inflammatory bowel disease (IBD) (44–46). In the rheumatoid synovium, ZFP36 is detected in macrophages, vascular endothelial cells, and fibroblasts (45). Interestingly, *ZFP36/TNF* gene expression ratio in synovial tissues correlates inversely with CRP (44). These findings suggest that inappropriate TTP production in response to increased TNF-α may be one factor that contributes to the pathogenesis of RA.

GWAS have also revealed that the *ZFP36L1* region is significantly associated with RA, JIA, Crohn’s disease, celiac disease, and type 1 diabetes (47, 48). In addition, *ZFP36L2* is identified as a susceptibility gene of MS, and its expression is decreased in MS patients compared to healthy subjects (49). Similarly, gene expression levels of *ZFP36L2* in peripheral blood mononuclear cells are significantly lower in SLE patients than those in healthy controls (50). Therefore, the variants of *ZFP36, ZFP36L1, and ZFP36L2* or dysregulation of those expressions may be involved in developing various inflammatory diseases in humans.

Regarding the association with allergic diseases, a comprehensive transcriptome analysis has shown that *ZFP36* expression in peripheral blood leucocytes is lower in persistent asthma children than in healthy children (51), suggesting that the reduction of *ZFP36* gene expression may be associated with asthma in children. Moreover, Leigh et al. have reported that budesonide inhalation induces various gene expressions including *ZFP36* in bronchial tissues and whole blood cells in healthy subjects (52), indicating that inhaled corticosteroids may provide anti-inflammatory effects by inducing *ZFP36* expression in both immune cells and non-immune cells. On the other hand, the expression of *ZFP36L1* in bronchoalveolar lavage cells is higher in patients with steroid-resistant asthma than that in patients with steroid-sensitive asthma (53). Hansel et al. have reported that *ZFP36L2* expression in peripheral blood CD4^+^ T cells is significantly higher in severe asthma patients than in mild asthma patients (54). Although further studies are required, these findings suggest that ZFP36 family proteins in immune cells and bronchial structural cells may contribute to the development of allergic airway inflammation and the sensitivity to inhaled corticosteroids.

## Clinical Potential of ZFP36 Family Proteins in Inflammatory Diseases

The forced expression of ZFP36 family proteins in peripheral tissues or immune cells could be novel therapeutic approaches for inflammatory diseases in humans (55). It has been reported that adenoviral overexpression of ZFP36 results in protection against bone loss and reduced inflammatory cell infiltration in experimental periodontitis in rats (56). Consistent with these findings, ZFP36-delta ARE mice, in which the stability of ZFP36 mRNA is enhanced by the deletion of a 136-base instability motif in the 3’ UTR of *ZFP36* mRNA, show the increased levels of ZFP36 expression in tissues (57) and are protected from anti-type II collagen antibody-induced arthritis, imiquimod-induced dermatitis, and experimental autoimmune encephalomyelitis (57). These findings suggest that sustained ZFP36 expression or activation is helpful in developing therapeutic strategies against inflammatory diseases.

As discussed in this review, the p38 MAPK pathway inactivates ZFP36 *via* the phosphorylation of two serine residues in mice and humans (20), while ZFP36 is dephosphorylated and activated by PP2A (58). Importantly, Ross et al. have reported that *in vivo* administration of PP2A agonists such as COG1410 (an apolipoprotein E peptide mimetic) or AAL(s) (a lipid derivative of the immunosuppressant FTY720 (Fingolimod)) activates ZFP36 through the dephosphorylation and ameliorates experimental murine arthritis models (45). Although the precise roles of ZFP36L2 in T cell function and inflammatory diseases remain to be elucidated, we have reported that ZFP36L2 reduces HELIOS expression in iTregs and suppresses iTreg function (**Figure 2**) (8). Thus, the reduction of ZFP36L2 expression in iTregs could be an attractive strategy for developing adoptive antigen-specific iTreg therapy.

## Concluding Remarks

RBPs, including ZFP36 family proteins, are essential for post-transcriptional regulation in RNA metabolism. Recent studies have uncovered that gene polymorphism of ZFP36 family members is associated with various autoimmune diseases and that the dysregulation of stabilization or inactivation by phosphorylation of ZFP36 family proteins could be involved in the pathogenesis of inflammatory diseases. However, it remains to be elucidated if there is functional redundancy and interaction among these family molecules for post-transcriptional regulation of immune responses. Therefore, a better understanding of the post-transcriptional processes mediated by each of the ZFP36 family members will be necessary to develop a novel therapeutic strategy for chronic inflammatory diseases.

## Author Contributions

SM, HT, and HN planned and wrote the MS. All authors contributed to the article and approved the submitted version.

## Funding

This work was supported in part by Grants-in-Aids for Scientific research from the Ministry of Education, Culture, Sports, Science and Technology (MEXT), LGS (Leading Graduate School at Chiba University) Program, Global Prominent Research (Chiba University), Takeda Science Foundation, and GSK Japan Research Grant.

## Conflict of Interest

The authors declare that the research was conducted in the absence of any commercial or financial relationships that could be construed as a potential conflict of interest.
